# Memorable Tourism Experiences in Red Tourism: The Case of Jiangxi, China

**DOI:** 10.3389/fpsyg.2022.899144

**Published:** 2022-07-05

**Authors:** Xuefei Zhou, Jose Weng Chou Wong, Shan (Anna) Wang

**Affiliations:** ^1^Jiangxi Vocational College of Finance and Economics, Jiujiang, China; ^2^Faculty of Hospitality and Tourism Management, Macau University of Science and Technology, Taipa, Macao SAR, China

**Keywords:** memorable tourism experience (MTE), country competence, place attachment, intention to visit similar destinations, special interest tourism (SIT), red tourism

## Abstract

Red tourism, as a form of special interest tourism (SIT), becomes widespread among Chinese tourists. This study aims to explore memorable tourism experiences (MTEs) in red tourism destinations and examines how country competence affects intention to visit similar destinations through the influences on MTEs, destination image, red tourism place attachment, and overall satisfaction. The partial least squares structural equation modeling (PLS-SEM) analysis is utilized to analyze the data from 556 tourists. Empirical results reveal that country competence positively influences MTEs and destination image. Furthermore, both MTEs and destination image influence overall satisfaction and red tourism place attachment, but do not influence intention to visit other similar destinations; the relationships between overall satisfaction, intention to visit other similar destinations, and place attachment are all confirmed as well. This study represents one of the few studies that classify red tourism as a form of historical-based SIT, with the purpose of promoting country competence. The outcomes provide a better understanding of both scholars and practitioners of historical-based SIT planning and businesses on how to promote destination image and country competence.

## Introduction

Red tourism is the tourism in which the national education of tourists through red tourism experience is carried out to achieve the political purpose and economic benefits of strengthening the governmental leadership (Xiao, 2013). Red tourism is not only the new emerging tourism pattern in Chinese destination but also a form of special interest tourism (SIT) (Tang et al., 2021), which indicate that tourists who travel to certain destinations pursue their special interests (Read, 1980).

Special interest tourism has been identified as a niche tourism strategy by governments around the world (Macleod, 2003). As Mckercher and Chan (2005) argued, in the SIT studies, examining why people visit a certain type of tourism destination is much more important than what they do there, destination management organizations can revise or develop their marketing strategy and promotion to better satisfy the tourists’ need. Previous studies of SIT have been focused on activity-based tourism such as Astro-tourism (e.g., Soleimani et al., 2019), food tourism (e.g., McKercher et al., 2008), and environmental-based tourism such as rural and urban tourism (e.g., Ma et al., 2020). However, there is a lack of SIT research that characterizes historical-based tourism as SIT from the demand side. Especially, it is argued that there is an emerging market for special interest tourists who want to study more about the history and culture of the national history (Light, 2000). Furthermore, while other historical-based tourism focuses mainly on the historical buildings or cultures, red tourism aims to show the country image and competence through the history of government leadership. However, existing package tourism products, namely, visiting cultural tours and museums, are often commercial and superficial (Witz, 2006); thus, these cannot fulfill the needs of special interest tourists who pursue the period of national history and enhance their country’s identity, such as the goal of red tourism. As a form of historical-based SIT with deep purposes, red tourism is the most significant tourism form to reflect the authentic history and has successfully attracted thousands of educating students, government officials, and other tourists in China (Poria et al., 2003; Timothy, 2011). Given that this kind of historical-based tourism with deep purposes is greatly overlooked in existing literature compared to commercial package historical tour, there is a need to extend our research to the historical-based SIT with deeper purposes, by considering the motives of red tourism.

As the competition among tourism destinations becomes more and more intense, there is a consensus that destinations need to seek ways to create memorable tourism experiences (MTEs) for tourists to increase their competitiveness (Neuhofer et al., 2012). MTEs are experiences that are selectively constructed from the entire tourism experience that can be remembered and recalled after the trip (Kim et al., 2012). When tourists visit a destination with a special interest, they participate in related activities that fulfill their needs (Akinci et al., 2016). The studies of Astro-tourism by Rodrigues et al. (2022) argued that SIT activities may lead people to generate MTEs. For tourists who are looking for a special interest, the factors that influence MTEs are consistently changing (Wong and Lai, 2021); thus, it is crucial to identify those factors leading MTEs in a certain form of SIT. Specifically, in the context of red tourism, since tourists’ experience of travel activities is influenced mainly by their perception of their national identification (Ali et al., 2016) and the destination image of that place (Hsu and Scott, 2020), MTEs may be associated with tourists’ perception toward their national identification and impression of the destination. Therefore, examining the effects between country competence, destination image, and MTEs helps answer the question of how this kind of historical-based SIT could be developed and leave tourists’ MTEs.

Referring to previous tourism studies, it is indicated that one destination providing tourists with MTEs can make tourists more satisfied (Chen et al., 2021), enhance their place attachment (Tsai, 2016), and increase their repeat behavior (Wong and Lai, 2021). This study embarks on an alternative way to test tourists’ behavioral intention of visiting other similar tourism destinations. Given that all forms of tourism are experience-based, it can be said that SIT, namely, red tourism, has its distinguishing characteristics, especially with regard to knowing about the different periods of national history (Zhao, 2020). Visiting one destination may provide tourists with MTEs and achieve their purpose of knowing history (Kim, 2014). However, red tourism usually consists of multiple routes, which represent different periods of history, and locates in different sites (Zhao, 2020). Thus, encouraging tourists to visit other similar destinations on red tourism routes can help the destination better promote historical-based SIT. In current studies, while most of the studies paid attention to tourists’ revisit intention, only very few studies considered tourists’ intention to visit other similar destinations (Wong et al., 2020), but it is crucial in some kinds of SIT, such as red tourism, as what they attempt to promote country competence by using the whole period of history with many destinations instead of only one destination. Therefore, this study fills the gap that discussing the efficiency and continuity of SIT because continuous traveling to other similar destinations is an important measure for tourism competitiveness and such behavior of continuous traveling would elicit more marketing strategies for tourism integrated development across regions (Wong et al., 2020).

The identified research gaps above drive this study to comprehensively inspect tourists’ intention to visit other similar red tourism destinations. More specifically, this study aims to clarify the relationship of the antecedent associated with red tourism and the influence of these variables on one another. The results of this study provide research contributions from four aspects, namely, (1) this study enriches the literature on SIT that red tourism (historical-based tourism) is classified as a type of SIT, based on the red tourists’ opinions in the aspects of the destination one appreciates and has interests. This information provides the identification of antecedents of visiting red tourism attractions. It offers a better understanding of SIT tourism planning in a global scope and guides tourism enterprises and government marketing strategies for destinations of showing the country competence. (2) This study links context-focused SIT with MTEs and contributes to the MTEs literature in SIT context. (3) This study offers a better understanding of the relationship between destination image and place attachment in the red tourism context. (4) It contributes to existing red tourism studies by revealing that visiting other similar destinations can be a more productive role in SIT. Practically, the results of this study provide insights for the relevant organizations, governments, and tourism stakeholders to develop red tourism destinations through a perspective of the country competence. To a larger extent, it gives implications for historical-based SIT development, especially in designing strategies for multiple tourism routes.

## Literature Review and Research Hypotheses

### Red Tourism

Red tourism is based on the revolutionary stories of the birth of China, the War of Resistance Against Japanese Aggression, the Chinese Civil War, and the founding of the People’s Republic of China (Wei et al., 2007). According to Li and Hu (2008), red tourism refers to “a theme tourism activity of learning, sightseeing and nostalgia in sites with a modern revolutionary legacy.” The main purpose of red tourism is to strengthen the government’s leadership, enhance national education and national identity of the public, and reduce regional development gaps (Caraba, 2011). Hence, in this study, the red tourism experience refers to tourists’ experience who travel the paths of iconic figures in the country history and can engage with, and in some cases re-enact episodes of, China’s government history. In addition, the red tourism experience includes multiple red activities and events, creating opportunities for people to participate in the revolutionary struggle as part of a broader program of nation building, so that people more deeply understand red culture. Combined with the characteristics of the red tourism experience, some connections between red tourism and historical-based heritage tourism are found since red tourism converts heritage into profitable tourism products, which uses the past to fulfill the present needs (Wall and Zhao, 2017). As Garrod and Fyall (2001) argued, historical-based heritage tourism, as a form of SIT, is a collection of everything that can be inherited, from historical buildings to works of art or beautiful landscapes. Different from normal heritage tourism with the purposes of only showing the tourism sites, history, and cultures, red tourism also aims to show the country image and competence through the history happening in red tourism destinations. It has been argued that this kind of heritage tourism has emerged as a particular form for special interest tourists (Light, 2000), which also reveals the connections between red tourism and SIT, because the special characteristics of red tourism provide special interest tourists with unique tourism experiences and fulfill their needs to know more about the period of history and understand the country *via* the government history.

The concept of SIT is generally defined as traveling for specific interests or motivations and providing a customized experience (Weiler et al., 1993; Douglas et al., 2001). SIT includes adventure, nature-based, religious, culinary, eco, culture and heritage, wildlife-watching, medical tourism, and so on (Weiler et al., 1993; Trauer, 2006; Kruja and Gjyrezi, 2011). It occurs when travelers’ motivation and decision-making are determined by a special interest with a focus on favorite activities or destinations, searching for a unique experience (Soleimani et al., 2019). In the red tourism setting, tourists who visit the red tourism destinations are motivated by its distinguishing characteristics (Zhao and Timothy, 2017) which dedicate to cultural and heritage aspects that motivate the trip to make individuals accessible to tourism and generate SIT around particular needs, such as understanding national history or building national identity. Red tourism can be understood in view of its heritage and historical and political background that shape activities and aroused the interest of the so-called red tourists. However, in many ways, red tourism appears to be different than heritage or cultural tourism. Much of the debates on heritage or cultural tourism are derived from the collection of tourism products, which aim to make profits such as historical building or activities that represent the stories of the past. However, red tourism also emphasizes the country’s great historical achievements and commemorative landmarks. The core of red tourism is to disseminate ideological education. Therefore, as a new emerging tourism pattern in China, red tourism is a type of tourism with specific features that are similar to but distinct from normal heritage or cultural tourism and can be described as SIT based on the common features with certain SIT typologies.

With the development of red tourism, theoretical research in this field is becoming more and more abundant, thus generates more research directions, such as marketing development of red tourism, national identity, and political role (Caraba, 2011; Zuo et al., 2017; Hung, 2018). Li et al. (2010) explored the interpretation of red cultural heritage by taking some famous red tourism attractions as examples; based on the destination image theory, Hunter (2013) combined the specific policies of red tourism context to define the basic principles of pilgrimage or religious tourism and visually analyze the image of Hunan’s network destinations and explore management methods; Zuo et al. (2016) linked tourism with the process of political socialization to analyze red tourism as the intermediary of political socialization effects and mechanisms in China, as well as the willingness of the ruling party to maintain its political power. However, the study of red tourism is still in its infancy and requires more representative research from tourists’ perspectives (Sima, 2017). The empirical studies of red tourism on how to attract special interest tourists pursuing to know more about national history are limited.

### Memorable Tourism Experience

Pine and Gilmore (1998) first proposed the “experience economy,” which indicates that only products and services are no longer to bring economic prosperity. Instead, companies are supposed to provide customers with a memorable “experience” (Pine and Gilmore, 2011). As tourism is an activity that can bring tourists different experiences (Tung and Ritchie, 2011; de Freitas Coelho et al., 2018), the industry is always devoted to bring positive feelings to tourists, and academic research on positive tourism experience has been increased. However, more professionals have realized that only making tourists satisfied during their travel experience is not enough. More importantly, they should consider how to enhance tourists’ memorability since it generates longer effects for tourism marketing (Wong and Lai, 2021). Tourism destinations need to increase their competitiveness by creating unforgettable experiences for consumers (Neuhofer et al., 2012). Reviewing the previous studies, MTEs is the closest concept to meet the “experience economy,” by emphasizing the importance of experience in the field of tourism (Fiske et al., 2002). MTEs refer to the experiences that are selectively built from the entire tourism experience, which can be remembered and recalled after the tour (Kim et al., 2012).

Kim et al. (2012) originally developed a seven-dimensional scale of unforgettable tourism experiences, including hedonism, novelty, local culture, refreshments, meaning, participation, and knowledge with 24 items. With the increasing attention to MTEs, many scholars have realized that MTEs should be applied to understand how tourists perceive their travel experience. Later, Kim (2018) further revised the dimensions of MTEs by simplifying previous items, which more comprehensively cover the concept of MTEs, and he used newly developed MTEs to examine their relationship with tourist’s satisfaction and revisit intention. In addition, some other scholars (Chen and Rahman, 2018; Wei et al., 2019; Gohary et al., 2020) also emphasized the importance of MTEs in examining tourists’ perception and future behavioral intention in different tourism settings, such as cultural tourism and eco-tourism. These studies all identified that MTEs have provided the insights into the tourism industry and are the key information to determine whether the tourists satisfy with the experience or decide to revisit a destination within a specific tourism type. However, MTEs on SIT are still underexplored and what actually motivates tourists to generate MTEs in SIT context is still unknown. Furthermore, as Benur and Bramwell (2015) stated, the measurement scales of MTEs may vary based on different tourism patterns, so it is necessary to revise the measurement scales of MTEs and test whether it is appropriate in SIT setting.

### Country Competence

Referring to previous studies, country competence refers to individuals’ identification with their country and it consists of wealth, economic development, modernization, and technology (Heslop et al., 2004; Nadeau et al., 2008; Zhang et al., 2016). Based on the agency theory, it has been found that country competence has a significant positive impact on the construction of initial trust (Jiménez and San Martín, 2010), and the establishment of initial trust makes it easier for consumers to accept relevant products and, to a certain extent, promotes consumer preferences for these products (Maher and Carter, 2011; Chen et al., 2014). Thus, consumers’ judgments on country competence are more likely to be linked to a country’s competence to provide high-quality products to generate trust and have a significant positive impact on product attitudes (Maher and Carter, 2011; Chen et al., 2014). Consistent with previous results, Diamantopoulos et al. (2017) used the stereotype content model to study the impact of explicit and implicit country stereotypes on consumer preferences and demonstrated that the country competence dimension drives consumers’ willingness to buy through the positive effects of branding (Maher and Carter, 2011; Halkias et al., 2016). In the tourism setting, country competence is examined in many studies from the perspective of a country’s image (Heslop et al., 2004; Zhang et al., 2016, 2018). Since the country image is generally defined as the overall impression, belief, and perception of the destination country (Roth and Diamantopoulos, 2009) and it includes the evaluation of the destination country’s history, geography, culture, politics, economics, and technology (Carneiro and Faria, 2016; Chaulagain et al., 2019), individuals with a higher level of country image will identify the country’s abilities.

As one of the important factors existing in the context of red tourism, which aims to strengthen national education and national identity (Caraba, 2011; Zhao and Timothy, 2017), country competence plays an important role on people’s identification of the country and it determines whether people will have MTEs during travel experience (Zhang et al., 2018). From the previous literature, it has been indicated that tourist’s perception of the country image significantly influences their travel experience. Zhang et al. (2018) found that the country image influences revisit intention through the mediating effect of their memorable travel experiences; Micevski et al. (2020) also identified that tourists’ emotion toward country competence is significantly related to the creation of an MTE. As a result, these studies all focus on the importance of country image and its relationship with tourist’s perception of the travel experience. Based on the results of the effects of country competence on the travel experience, Zhang et al. (2018) developed a measure for country image in an international tourism setting. They indicated that the higher the tourist’s perception of the country competence, the more positive travel experience tourists have. This study extends the country competence in the red tourism context. For red tourists, their perception of a country’s political, historical, and environmental aspects probably has an influence on their travel experience. Thus, we have proposed that tourists with a higher perception of their country competence will have more positive MTEs in red tourism destinations, and the following hypothesis is presented.

*H1a: Country competence is positively related to MTEs.*

### Destination Image, Place Attachment, and Overall Satisfaction

Destination image is an important aspect of influencing tourists’ decision-making, destination choice, and future behavior (Stylos et al., 2016; Zhang et al., 2016; Afshardoost and Eshaghi, 2020). Agapito et al. (2013) defined the image of a destination as a person’s perception of the destination’s attributes and the overall impression of the destination individual. The destination image refers to the image of the core tourism products directly related to tourist attractions and tourist facilities that directly meet the core tourist needs (Zhang et al., 2016). According to the product-country image (PCI) framework (Roth and Diamantopoulos, 2009), if the destination is regarded as an experienced product, the perception of the country image will affect the image of this product (Wang et al., 2012). Thus, individuals’ perception of the country has a significant effect on the destination image (Zhang et al., 2016, 2018). It is indicated that the higher level of individuals’ identification of the country, the better the destination image is. Zhang et al. (2018) also illustrated that country competence significantly affects destination image. In a recent study, Kim (2014) further explored the destination image associated with MTE and supported the relationship. It is indicated that destination image is an important factor for the MTEs (Chen and Tsai, 2007; Lu et al., 2015; Zhang et al., 2018). It is generally agreed that destination image depends on attribute-based destinations that create an overall impression. Many destination-level attributes contribute to the evaluation of destination impression (Hahm et al., 2018). Based on previous literature, people’s perception of country competence has been found to influence individuals’ destination image formation. Tourists perceived a better destination image in a tourism area, and they can be more concentrated on this tourism experience and further make this experience unforgettable. Thus, the following hypotheses are drawn.

*H1b: Country competence is positively related to destination image.*

*H2a: Destination image is positively related to MTEs.*

Place attachment is the connection between an individual and a particular environment (Lee et al., 2012). Extended the concept of place attachment from Low and Altman (1992), red tourism place attachment refers to one tourist’s interplay of emotion, knowledge, and belief with respect to the red tourism destination. From previous studies, the role of place attachment has been identified. For example, Veasna et al. (2013) established and validated a comprehensive theoretical model of destination image, and SEM analysis found that the destination image does have a significant positive impact on place attachment (Prayag and Ryan, 2012). As destination image is related to the subjective feelings and overall evaluation of a particular destination (Bigné et al., 2001; Wang et al., 2020), it is the decisive factor influencing visitors’ attitude toward the destination (Prayag and Ryan, 2012). When tourists perceive a good level of perception of the destination image, the meaning of the trip may be enhanced; thus, they may have an attachment to the destination. According to Chen (2018), destination image can be understood as the determinant factor of place attachment in film tourism. Similarly, Chiang (2016) supported the relationship between destination image and place attachment in the night market context. From the above literature, there is a general agreement that individuals’ emotional connection toward a destination should be explained through tourists’ perceived image of the destination. The reason is red place attachment is the emotional reaction toward the red experience, while destination image describes the cognitive or affective aspects of the tourism destination. This relationship remains unexplored in the context of red tourism. Therefore, this study proposes that a more favorable destination image will generate people’s stronger place attachment to red tourism and the following hypothesis is presented:

*H2b: Destination image is positively related to red tourism place attachment.*

Tourism satisfaction refers to “the discrepancy between the initial expectation and the perception after travel experience” (Oliver, 1980). Research shows that there is a significant positive impact between tourism destination image and satisfaction (Prayag and Ryan, 2012; Loi et al., 2017; Kim, 2018). When tourists have a good evaluation after experiencing the destination, they will deeply enjoy the trip and have a better overall satisfaction. This is further supported by Lam et al. (2020), who proposed that tourist satisfaction largely depends on travel destination image. As such, it can be expected that the destination image of red tourist destination will influence satisfaction in this study, and the following hypothesis is proposed:

*H2c: Destination image is positively related to overall satisfaction.*

Tsai (2016) proposed that MTEs have a significant positive impact on place attachment. The more memorable red tourism experience means the more meaningful the tourism experience tourists gain; thus, they are more likely to get an attachment to that place. Such a positive tourism experience can determine tourist attachment to a destination, which is supported by Vada et al. (2019) in the cultural tourism setting. In addition, MTEs also show a significant effect on overall satisfaction (Prayag and Ryan, 2012; Kim, 2018; Wong et al., 2019). More specifically, Chen et al. (2021) found that an MTE on coffee tourism strongly reinforced tourists’ satisfaction. Stavrianea and Kamenidou (2021) revealed that the formation of tourists’ satisfaction is greatly related to positive MTEs. Therefore, once the red travel experience has more factors that make tourists unforgettable, they are more likely to enjoy the entire trip and be satisfied. The following hypotheses are proposed in the red tourism context:

*H3a: MTEs are positively related to red tourism place attachment.*

*H3b: MTEs are positively related to overall satisfaction.*

Previous research has suggested that place attachment is a predictor of satisfaction and revisit intention (Prayag and Ryan, 2012; Ramkissoon et al., 2013; Abou-Shouk et al., 2018). In addition, Abou-Shouk et al. (2018) proposed that place attachment has a positive relationship with overall satisfaction. When a special emotional connection can be created between the tourist and the destination, this type of destination has special significance for the tourist, and it is easier for the tourist to enjoy and immerse in this experience. Therefore, it is more likely that the overall satisfaction of tourists will increase with place attachment of red tourism destinations and the following hypothesis is proposed:

*H4a: Red tourism place attachment is positively related to overall satisfaction.*

### Intention to Visit Other Similar Destinations

Revisiting intention refers to the intention of visitors to return to a place and it is widely used to examine tourists’ satisfaction (Um et al., 2006). The tourism industry has recognized the importance of revisit intention because the destination gains reputations such as word of mouth and brand loyalty through revisit customers (Petrick, 2004). Therefore, willingness to revisit is very important for operators in the tourism industry (Shonk and Chelladurai, 2008). However, tourists always want to explore new destinations (Barros et al., 2011; Zhang et al., 2018), and although they are very happy and satisfied with a particular destination, they may not return to the same destination to experience the same activities (Oppermann, 2000). Breiby and Slåtten (2018) divided the concept of the intention of revisit into revisiting this specific route and visiting other similar routes. Stepchenkova et al. (2015) found that tourists’ intention to revisit not only exists in the material exploration of specific historical buildings but also exists in the exploration of other similar experiences. Visiting other similar destinations can both fit tourists’ interests and help tourists to explore new experiences. As for red tourism, which is based on long-term history and involves many heritage destinations, tourists may prefer to visit different red destinations to understand the national culture more completely and deeply. Thus, the intention to visit similar destinations is much more meaningful to study in the current context. According to Wong et al. (2020) concept of visiting similar destinations, this term refers to visiting other destinations with similar red tourism characteristics. The evidence from previous studies has shown that the nature of revisit intention is determined by several factors, such as satisfaction, destination image, MTEs, and place attachment (Wang and Hsu, 2010; Abou-Shouk et al., 2018; Kim, 2018). More specifically, the study proposed by Cham et al. (2021) on 600 tourists found that the image of Malaysia is positively associated with their intention to revisit the destination; Wong et al. (2020) explained that visiting other similar destinations is determined by MTEs. Similarly, some scholars (Brown et al., 2016) also emphasized that both place attachment and satisfaction can help to explain tourists’ revisit intention. Accordingly, when the destination image and the tourism experience are memorable, the meaning of the tour will be enhanced, and the tourist may be attached to this kind of destination, which may prompt them to other similar destinations. Therefore, the following hypotheses are raised:

*H2d: Destination image is positively related to the intention to visit other similar destinations.*

*H3c: MTEs are positively related to the intention to visit other similar destinations.*

*H4b: Red tourism place attachment is positively related to the intention to visit other similar destinations.*

*H5: Overall satisfaction is positively related to the intention to visit other similar destinations.*

## Methodology

### Variable and Measurement

**FIGURE 1:**
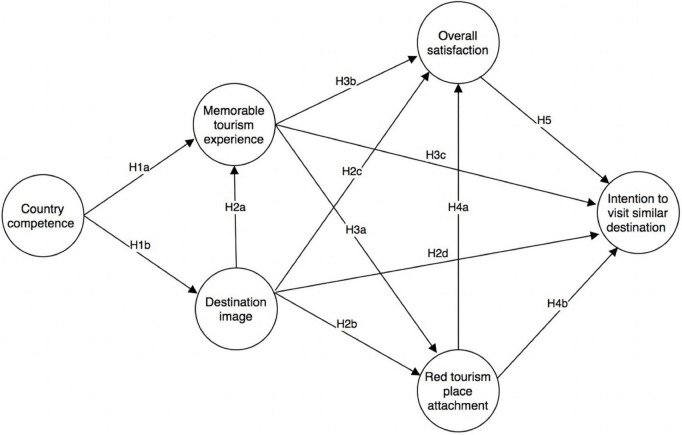
Conceptual model.

The main purpose of this study is to explore the antecedents and intermediaries of MTEs in the context of red tourism (Please see the Figure 1 for the proposed conceptual model). Jiangxi province in China is a representative case study in terms of the degree of development and theoretical research. According to the National Red Tourism Development Planning 2004–2010, which was publicized jointly by the State Council and the Central Committee of CCP, red destinations are distributed across the whole regions of China, so that tourists have the chance to visit other similar red destinations after the tour. Jiangxi province is the cradle of the Chinese revolution and a revolutionary resort, because a series of major revolutionary activities took place here and many red resources are well preserved. According to the report released by the Jiangxi Tourism Planning and Research Institute, red tourism has attracted 143 million tourists, accounting for one-eighth of the national red tourism tourists, while the national red tourism comprehensive income accounts for more than one-third of the total in 2016. The histories of red tourism destinations are meaningful for tourists. Therefore, the questionnaires were distributed to the three most popular sites in the famous red tourism destination, i.e., Jinggangshan.

This questionnaire included six constructs. All questions adopted a seven-point Likert scale ranging from “very disagree” to “very agree.” The scales of each measurement were adapted from previous literature and slightly modified to fit the context of red tourism. The measurement of country competence was borrowed from Zhang et al. (2018) with four items. Six items from Kim (2018) and Prayag and Ryan (2012) were utilized to measure destination image. The measurement scale of MTEs was assessed by Kim (2018) with five items. Six items were revised and employed to measure place attachment from Scarpi et al. (2019). For these two constructs, some of the key terms like “tourism experience” and “culture” were slightly revised to “red tourism experience” and “red culture” to reflect the contexts of this study. Referring to previous studies, this study measures tourists’ overall satisfaction in three items that were adapted from Kastenholz et al. (2018). The construct of intention to visit similar destinations was assessed by Kim (2018). To fit this study, some adjustments were slightly made to the revisit intention scale.

The measurement items were originally written in English that translated into Chinese by professional translators. To ensure that all questions were easy to understand and unambiguous, a pilot test was conducted with 20 postgraduates who are majoring in tourism management with red tourism experience. The questionnaire was slightly modified according to their opinions. Table 1 shows the measurement items of this study.

**TABLE 1:** Assessment of the measurement model and descriptive statistics.

Items	Concepts	α	CR	Loading	AVE	R^2^
**Country competence**	It refers to Individuals’ identification with their country (Zhang et al., 2016).	0.876	0.915		0.729	
China is a wealthy country				0.836		
China has advanced economy development				0.856		
There is a high level of modernization in China				0.871		
China has advanced technology				0.851		
**Destination image**	The perception of the destination’s attributes and the overall impression of the destination (Zhang et al., 2016).	0.853	0.891		0.577	0.220
The destination image of quality of service				0.797		
The destination image of entertainments				0.800		
The destination image of quality and variety of accommodations				0.749		
The destination image of local transportation				0.755		
The destination has red tourism image				0.736		
The image of architectures/buildings at the destination				0.716		
**Memorable tourism experiences**	The experiences that are selectively built from the entire tourism experience, which can be remembered and recalled after the tour (Kim et al., 2012).	0.856	0.897		0.635	0.540
I really enjoyed this red tourism experience				0.811		
I revitalized through this red tourism experience				0.834		
I learned something about myself from this red tourism experience				0.819		
I had a chance to closely experience the local red culture of a destination area				0.781		
I experienced something new (e.g., food activity, etc.) during this red tourism experience				0.736		
**Red tourism place attachment**	It refers to one tourists’ interplay of emotion, knowledge and belief with respect to the red tourism destination (Low and Altman, 1992).	0.880	0.909		0.625	0.628
I enjoy participating in red tourism more than any other				0.808		
No other can compare with red event				0.832		
This place is the best place for red events				0.776		
I am very attached to red tourism				0.760		
This red tourism means a lot to me				0.785		
I feel like this red tourism is part of me				0.780		
**Overall satisfaction**	The discrepancy between the initial expectation and the perception after travel experience (Oliver, 1980).	0.891	0.932		0.821	0.626
I feel enjoyable about this tourism experience				0.899		
I feel pleasant about this tourism experience				0.921		
I am satisfied with this tourism experience				0.897		
**Intention to visit similar destination**	The intention to visit a place with similar cultural characteristics (Wong et al., 2020).	0.844	0.906		0.763	0.441
I would like to other red destination in a year				0.877		
I plan to other red destination in a year				0.898		
I will make an effort to other red destination in a year				0.845		

### Sample and Data Collection

Data were collected from December 2021 to January 2022 by three well-trained research assistants at the main entrances of three red tourism attractions of Jinggangshan. The systematic sampling method was employed because this method leads to a more representative survey, as suggested by Elsayir (2014). A random number “5” was selected, and every fifth tourist was invited to participate in the survey. If the selected tourist was not appropriate for the survey (e.g., under 18 years or decline to participate), the next person was subsequentially selected to fill out the survey. All research assistants strictly followed the prevention policies of COVID-19 by wearing masks and keeping a distance to take the survey. All respondents answered the questionnaire with their consent and fully voluntarily participated. As a result, a total of 580 questionnaires were collected in this study, 24 of which were removed from the survey as these contain incomplete questions or consistent similar scores (e.g., all 7 or all 1). There were 556 valid samples left finally with a 96% useful rate. The valid samples are considered sufficient for further data analysis, as Hair et al. (2021) recommended the sample size of 1:10. Details of the sample are presented in Table 2.

**TABLE 2:** Details of sample responses (*N* = 556).

Details	Frequency	Percentage (%)
**Gender**		
Female	284	51.1
Male	272	48.9
**Age**		
18–20	99	17.8
21–25	98	17.6
26–30	52	9.4
31–35	52	9.4
36–40	54	9.7
41–45	44	7.9
46–50	39	7.0
51–55	55	9.9
56–60	34	6.1
61 years and above	29	5.2
**Education**		
≤Middle school	64	11.5
High School	64	11.5
Bachelor	340	61.2
Master Degree	74	13.3
≤PhD.	14	2.5
**Distribute**		
Local	125	22.5
Inside the province	277	49.8
Other province	154	27.7

## Results

Descriptive statistical analysis was performed using SPSS version 2.1. Moreover, to test the proposed hypothesis, partial least squares structural equation modeling (PLS-SEM) was employed using SmartPLS version 3.0 (Roldán and Sánchez-Franco, 2012; Ringle et al., 2015). The rationale is PLS-SEM helps to predict the dependent variables and explain the complex models or relationships; in addition, PLS-SEM can maximize the variance of dependent variables and require a small to medium sampling size (Hair et al., 2017).

### Measurement Model

The reliability, convergent, and discriminant validity of reflective constructs were assessed by the following previous PLS-SEM research (Hair et al., 2010). Composite reliability (CR) value of 0.6 or more is acceptable, and the higher the value, the better the internal consistency of the variable (Hair et al., 2021). Cronbach’s alpha (α) has an acceptable reliability score of 0.7 or higher (Heale and Twycross, 2015). In addition, factor loading is more than 0.7, and average variance extracted (AVE) is over 0.5 threshold values (refer to Table 1). Moreover, the square root of AVE is larger than any other correlation among constructs (refer to Table 3). Thus, the reliability and convergent and discriminate validity were ensured.

**TABLE 3:** Construct intercorrelations.

	CC	DI	IVSD	MTEs	OS	PA
CC	**0.854**					
DI	0.469	**0.759**				
IVSD	0.385	0.511	**0.873**			
MTEs	0.430	0.728	0.533	**0.797**		
OS	0.361	0.703	0.572	0.714	**0.906**	
PA	0.453	0.754	0.645	0.717	0.731	**0.791**

*Numerical value in bold is the square root of AVE. IVSD = intention to visit similar destination; CC = country competence; PA = red tourism place attachment; OS = overall satisfaction; DI = destination image; MTEs = memorable tourism experiences.*

### Structural Model

**FIGURE 2:**
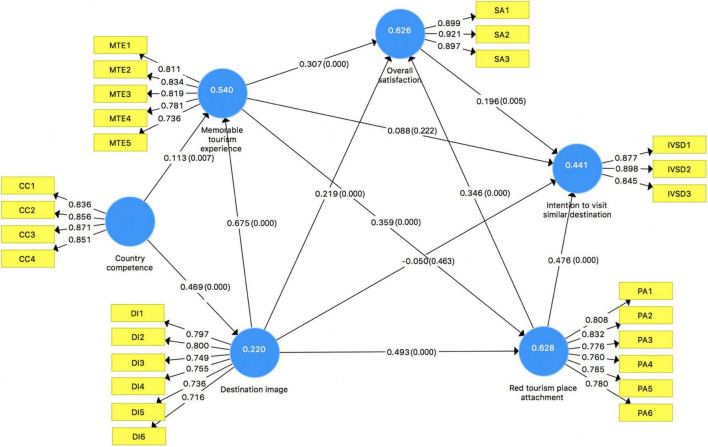
Result of PLS-SEM analysis.

The results of the PLS-SEM analysis are shown in Figure 2, Tables 4, 5. R^2^ value is greater than 0.10 and closer to 1, indicating that the more variance is explained between the latent variable, the greater the degree of influence of this factor (Hair et al., 2017). In this study, all constructs met the threshold (refer to Table 1). Thus, this proposed model is well explained and is highly predictive. A p-value is less than 0.05, and the path coefficient is significant (Gentle et al., 2012). In the study, except for H2d and H3c, all other paths had a significant structural path coefficient (*p* < 0.001). The results of PLS-SEM indicate that country competence and destination image had significantly direct impact on MTEs at 0.001 confidence level (β = 0.113, *p* = 0.007; β = 0.675, *p* = 0.000). Therefore, H1a and H2a were supported, respectively. Results also showed that country competence had a significantly positive effect on destination image (β = 0.469, *p* = 0.000), which supported H1b. Similarly, the effect of destination image (β = 0.493, *p* = 0.000) and MTEs (β = 0.359, *p* = 0.000) on red tourism place attachment was significant and positive, which provided support to H2b and H3a. Consistent with H2c, H3b, as well as H4a, destination image (β = 0.219, *p* = 0.001), MTEs (β = 0.307, *p* = 0.000), and red tourism place attachment (β = 0.346, *p* = 0.000) were found to be positively related to overall satisfaction. Similarly, place attachment (β = 0.476, *p* = 0.000) and overall satisfaction (β = 0.196, *p* = 0.005) were found to be positively related to intention to visit other similar destinations. However, it indicated that overall satisfaction had a significant direct impact on intention to visit other similar destinations at a 0.005 confidence level. Thus, H4b and H5 were supported.

**TABLE 4:** Summary of hypotheses’ test results.

Hypothesis	Coefficient	P-value	Accept/Reject
H1a: Country competence → MTEs.	0.113	0.007	Accept
H1b: Country competence → Destination image	0.469	0.000	Accept
H2a: Destination image → MTEs	0.675	0.000	Accept
H2b: Destination image → Red tourism place attachment	0.493	0.000	Accept
H2c: Destination image → Overall satisfaction	0.219	0.000	Accept
H2d: Destination image → Intention to visit other similar destination	–0.050	0.463	Reject
H3a: MTEs → Red tourism place attachment	0.359	0.000	Accept
H3b: MTEs → Overall satisfaction	0.307	0.000	Accept
H3c: MTEs → Intention to visit other similar destination	0.088	0.222	Reject
H4a: Red tourism place attachment → Overall satisfaction	0.346	0.000	Accept
H4b: Red tourism place attachment →Intention to visit other similar destination	0.476	0.000	Accept
H5: Overall satisfaction → Intention to visit other similar destination	0.196	0.005	Accept
			

**TABLE 5:** Effect deconstruction of structural model (standardized value).

Explanatory variable	Explained variable														
	
	DI			MTEs			PA			OS			IVSD		
					
	DE	IE	TE	DE	IE	TE	DE	IE	TE	DE	IE	TE	DE	IE	TE
CC	0.469	–	0.469	0.113	0.317	0.430	–	0.385	0.385	–	0.368	0.368	–	0.270	0.270
DI				0.675	–	0.675	0.493	0.242	0.734	0.219	0.461	0.680	–	0.543	0.493
MTEs							0.359	–	0.359	0.307	0.124	0.431	–	0.255	0.343
PA										0.346	–	0.346	0.476	0.068	0.544
OS													0.196	–	0.196

*DE: direct effect, IE: indirect effect, TE: total effect. IVSD = intention to visit similar destination; CC = country competence; PA = red tourism place attachment; OS = overall satisfaction; DI = destination image; MTEs = memorable tourism experiences.*

### Direct, Indirect, and Total Effects

As for the mediating effect among the constructs, the study showed the direct, indirect, and total effects among variables that are explanatory and explained in Table 5. For MTEs, destination image showed a higher level of a direct effect than country competence (0.113 and 0.675, respectively). In addition, for the intention to visit a similar destination, place attachment shows a better effect than overall satisfaction. Furthermore, place attachment and overall satisfaction completely mediated the relationships among MTEs, destination image, and intention to visit similar destinations.

## Discussion and Conclusion

This study aims to explore the factors that influence MTEs and present a comprehensive theoretical model. Through the hypothesis testing of the proposed model, it was found that ten hypotheses were accepted. First, perceived country competence and destination image had a positive effect on MTEs. The higher the evaluation of the ability of the destination country, the more positive the evaluation of the destination image and the greater the possibility of generating MTEs during the destination. As Zhang et al. (2018) argued, MTEs are the best predictors of future destination choices, while destination attributes are important antecedents of MTEs (Kim, 2014). The findings of this study supported this argument. In addition, the coefficient of destination image is higher than country competency on the impact of MTEs. It may be caused by the following two possible reasons: one is that country competence may be only a part of the country image, another is that the perception of country competence may differ depending on the degree of their national identity or their curiosity of the country.

Second, red tourism place attachment and overall satisfaction are fully mediating among destination images, country competence, and the intentions to visit similar destinations. That is not consistent with previous studies (Prayag and Ryan, 2012; Coudounaris and Sthapit, 2017; Chen and Rahman, 2018; Kim, 2018; Zhang et al., 2018). It may be because the MTEs generated by tourists and the perception of the destination image are both created by this destination at the same time. Unless these MTEs or destination images can create an emotional dependence between the visitor and the destination, making the destination meaningful for the tourists. Then, they may be willing to travel to similar destinations. Consistent with previous studies about place attachment (Tsai, 2016), the unique local experiences enable tourists to create MTEs, and such MTEs further enhance the identity or strong attachment of tourists to local attractions and behavioral intentions.

Third, red tourism place attachment has a more significant impact on the intention to visit similar destinations than overall satisfaction. Inconsistent with the results of this study, Abou-Shouk et al. (2018) found that tourists’ satisfaction is far more positive than place attachment to revisit intentions. Similarly, this may be because satisfaction is a targeted and time-sensitive emotion. Although it may prompt tourists to revisit the same destination, they may not be able to continue to a similar one. Therefore, although the tourism experience makes tourists more satisfied, it does not necessarily encourage tourists to tourism to the same type of destination. In conclusion, this study contributes to SIT research by expanding the SIT empirical study in a form of historical-based tourism and fills the gap by connecting SIT with the MTEs framework. A further discussion of theoretical and practical implications is presented below.

### Theoretical Implication

Red tourism, as a form of SIT, attracts tourists who are motivated by their special interest of the country history. In the SIT research, most researchers have focused on special forms of activities or unique environments in the tourism destination but overlooked the special interest of history-based tourism from a national perspective. This study represents a step forward in the efforts to characterize red tourism as a type of SIT, thus theoretically contributing to SIT literature. Moreover, it shows that country competence is the key factor in the configuration of the red tourism context. Country competence, as one of the representative signals of national identity and pride, plays an important role in red tourism. It provides a better understanding that the role of country competence should be considered in the historical-based SIT context with a global scope. With different kinds of historical-based SITs all around the world, researcher may further consider what in-depth factors, like country competence, may help explore the meanings and characteristics of the historical-based SIT, instead of taking heritage tourism as normal sightseeing places or commercial tourism products.

Second, this study has successfully applied the MTEs framework in the SIT context with other constructs on tourist overall satisfaction, red tourism place attachment, and intention to visit other similar destinations. For tourists’ satisfaction, MTEs show the strongest effects on it, which confirmed Wong and Lai’s (2021) arguments that tourists’ satisfaction greatly depends on their positive emotional satisfaction. Previous studies on MTEs mainly focus on destination management and marketing. This study may be one of the first empirical studies to consider MTEs in a certain form of SITs. It implies that memorable experience has become a key influential factor for SIT overall experience, particularly with respect to the role of country competence in evaluating what constitutes a memorable experience. The SIT market has been investigated continuously and becomes the dominant force that refines different types of new emerging tourism types. This study provides insights for further studies to consider developing MTEs in other forms of SIT.

Third, this study is necessary for a better understanding of the relationship between destination image and place attachment in the red tourism context. Red tourism place attachment is an important component in understanding the close relationship between a tourist and red tourism destinations, but it is also formed by destination image. The good impression of the destination aroused their sense of identity with red tourism places and felt that there were some connections because the destination may enhance their perceptions to red history. Many studies have addressed the influence of destination image on place attachment (Chen, 2018). However, this study emphasizes the line from a positive destination image to attachment to a specific tourism pattern.

Fourth, this study highlights the important role of the tourists’ perceptions that influence tourists to consider further red tourism trips in other destinations. Most of the existing studies widely addressed the importance of repeat behavior and have put revisit intention as tourists’ behavioral outcome (Zhang et al., 2018; Rather, 2021; Soliman, 2021). However, some tourism patterns may spread in multiple destinations, which have connections and represent a period of history, such as the revolutionizing tourism of the Silk Road (Shehzad et al., 2019). For such regions, it is necessary that tourists like the specific tourism pattern and keep traveling to other similar destinations. Thus, this study argues that SIT should focus not only on revisit intention but also on the consistent travel pattern, which makes tourists intend to visit other similar destinations in one region.

### Implications in Practice

As for practical contributions, this study provides guidelines for different stakeholders. First, tourism operators should take country competence as a priority since it is a direct factor influencing tourists’ perceptions and behavioral intentions in red tourism. The destination managers should put more efforts into making tourists aware of the national development and competence. For example, during the visit, the attraction sites may consider providing areas or public screens for presenting the historical storyline by a short video. The purpose of presenting short videos is to help tourists understand how China grew from a poor country to the present, thus, increasing their pride, confidence, and identification to the nation. In addition, new technologies can be applied (e.g., augmented reality or virtual reality) to provide tourists’ opportunities to truly experience the history, which enhances their understanding of the country competence. In contrast, Maher and Carter (2011) and Chen et al. (2014) and suggested that the way to increase people’s national identification is by using national brand products. Thus, the destination marketing may invest more in national brand products, such as food and beverage, fashion products, and souvenir.

Second, the success of managing red tourism or other historical-based SIT should deliver a high level of destination image, because it eventually contributes to red tourism place attachment. Destination marketers should focus on the branding strategy of one destination. For example, when tourists visit a red tourism attraction, all the facilities and equipment (e.g., decoration, table and chairs, and lodging) should be designed in a consistent vintage style with red tourism characteristics. When tourists recall the travel experience, they will have a higher level of destination image with red tourism characteristics and form better MTEs. Understanding this link can help destination management to develop a long-lasting bonding with tourists and more effectively retain their visitors within other kinds of historical-based destinations.

Third, according to the results, place attachment is the most important dimension influencing a tourist’s future behavior. It suggests that the connection between individuals and destination should be enhanced, because red place attachment includes people’s psychological attachment based on history or built through experiences (Zhang et al., 2021). Destination marketing should better design a variety of leisure and interactive activities based on different characteristics of red tourism destinations to encourage people’s participation, which further enhances their sense of belonging to the destination. For example, the destination can promote a history quiz with prizes, so that the winners get their free photos taken with families in the red tourism destination. In addition, tourists can learn to sing an old song during the red history period to get souvenirs with red tourism characteristics. In contrast, when developing activities, destinations should avoid over-commercialization in red tourism destinations and strengthen the supervision and protection of such historical-based special tourism destinations, in order not to affect tourists’ destination image and place attachment.

Fourth, from the social perspective, this study argues that SIT like red tourism is worth examining and it provides a comprehensive framework to attract tourists and influence tourists’ behavioral intentions. It is valuable for the local residents, organizations, and society to understand the core values of the red tourism destinations in their hometown. Especially, many of the red tourism destinations are relatively poor in terms of resources. Understanding the core values of red tourism would help them understand the tourists and better support the sustainable tourism development.

Finally, from a government perspective, they should enhance the cooperation between various red destinations. More clearly, in order to better promote the historical-based SIT, the strategy should focus on how to encourage tourists continuously visit other similar destinations. Destination managers and governments from different cities should work together to attract more people to visit this form of SIT. For example, the nearby red tourism attractions may promote the ticket or tourism package to attract tourists to visit several red tourism sites in 1 year. In addition, destination management organizations should put efforts on advertising other red tourism destination routes. For example, they should provide information about red tourism sites in other cities for existing tourists.

### Limitation and Future Study

It is important to note that there are several limitations that may provide directions for future research. First, this study examined the conceptual model with the data from tourists in Jiangxi, China, which has limitations in the composition of samples. Future studies can be performed from the perspectives of residents and include more diverse red tourism attractions. Second, the study identified country competence as the antecedent of red tourism motives. Future studies are recommended to investigate other factors that motivate people to visit historical-based SIT tourism sites (e.g., perceived value, tourists’ emotion, and national education). Third, this study only focused on the direct relationships between constructs. Future studies may examine some potential moderating effects. Fourth, considering that this study emphasized the red tourism as a form of historical-based SIT, it has limitations on measurement item generalization in other historical-based SIT research. Future studies should include other historical-specific aspects when developing the measurement items.

## Data Availability Statement

The raw data supporting the conclusions of this article will be made available by the authors, without undue reservation.

## Ethics Statement

Ethical review and approval were not required for the study on human participants in accordance with the local legislation and institutional requirements. Written informed consent from the patients/participants’ legal guardian/next of kin was not required to participate in this study in accordance with the national legislation and the institutional requirements.

## Author Contributions

All authors listed have made a substantial, direct, and intellectual contribution to the work, and approved it for publication.

## Conflict of Interest

The authors declare that the research was conducted in the absence of any commercial or financial relationships that could be construed as a potential conflict of interest.

## Publisher’s Note

All claims expressed in this article are solely those of the authors and do not necessarily represent those of their affiliated organizations, or those of the publisher, the editors and the reviewers. Any product that may be evaluated in this article, or claim that may be made by its manufacturer, is not guaranteed or endorsed by the publisher.
